# Staying moving, staying strong: Protocol for developing culturally appropriate information for Aboriginal people with osteoarthritis, rheumatoid arthritis, lupus and gout

**DOI:** 10.1371/journal.pone.0261670

**Published:** 2021-12-23

**Authors:** Penny O’Brien, Brooke Conley, Samantha Bunzli, Jonathan Bullen, Juli Coffin, Jennifer Persaud, Tilini Gunatillake, Michelle M. Dowsey, Peter F. Choong, Ivan Lin

**Affiliations:** 1 Department of Surgery, St Vincent’s Hospital Melbourne, The University of Melbourne, Melbourne, VIC, Australia; 2 Curtin Medical School, Curtin University, Perth, WA, Australia; 3 Telethon Kids Institute, Broome, WA, Australia; 4 Arthritis and Osteoporosis Western Australia, Perth, WA, Australia; 5 Physiotherapy Department, Sir Charles Gairdner Hospital, Perth, WA, Australia; 6 Western Australian Centre for Rural Health, The University of Western Australia, Geraldton, WA, Australia; 7 Geraldton Regional Aboriginal Medical Service, Geraldton, WA, Australia; University of South Australia, AUSTRALIA

## Abstract

**Introduction:**

Addressing disparities in arthritis care is an important yet unmet health need for Aboriginal and Torres Strait Islander people in Australia (respectfully Aboriginal people herewith). Despite the significant prevalence and burden of arthritis within Aboriginal communities, access to care for arthritis is low. One means to reduce existing disparities in health care is to address current challenges relating to the appropriateness and acceptability of health care information resources for Aboriginal people. Health information sources can help to empower patients and their families to have greater involvement in their care and to engage in self-management of their condition. Despite an extensive range of arthritis information resources being available, currently no resources have been culturally adapted and developed in collaboration with Aboriginal consumers with arthritis. This paper outlines the processes that will be undertaken within the *Staying Moving*, *Staying Strong* project. This project aims to develop culturally secure arthritis information for Aboriginal people with osteoarthritis, rheumatoid arthritis, lupus and gout.

**Methods and analysis:**

The overarching principle guiding this project is cultural security, referring to the incorporation of processes such that the research will not compromise the cultural rights, values and expectations of Aboriginal people. This project will prioritise partnerships, community engagement, community benefit, sustainability, transferability, and capacity building and therefore uphold the cultural rights and values of Aboriginal people. In this six-phase project we will; 1) Establish a community reference group and advisory committee; 2) Explore the health information needs and preferences of Aboriginal people with arthritis; 3) Synthesise the existing key recommendations in high quality clinical practice guidelines on arthritis care; 4) Culturally adapt key clinical recommendations; 5) Develop culturally appropriate arthritis resources and; 6) Qualitatively evaluate the developed resources.

## Introduction

Ensuring timely access to equitable, high quality health care for all populations within society is an integral component of the right to health and is a goal of Australia’s universal health care system [1, 2]. Barriers to access arise within health care when cultural factors are not acknowledged, respected and prioritized [3]. Cultural security describes a commitment to providing health care services that do not compromise the cultural rights, values, beliefs, knowledge systems and expectations of Aboriginal and Torres Strait Islander people (respectfully Aboriginal people herewith) and must be considered in the development and delivery of meaningful patient education and information resources [3, 4].

Addressing the burden associated with musculoskeletal conditions experienced by Aboriginal people is an emerging priority in Aboriginal health care and research [5–7]. The Australian Department of Health National Strategic Action Plan for Arthritis recommends providing information, education and support to people with arthritic conditions so that they can effectively self-manage their symptoms [8]. This includes prioritising the development of comprehensive health promotion resources and expanding and tailoring existing resources for Aboriginal people [8]. Few culturally adapted health information resources have been designed to meet the needs of Aboriginal people living with arthritis and those that have been developed have not been robustly evaluated [9]. This study aims to address these issues by reviewing existing patient information resources available for arthritic conditions, including osteoarthritis, rheumatoid arthritis and systemic lupus erythematosus (lupus) and gout. Working in partnership with Aboriginal community members, Aboriginal health researchers, and arthritis experts, we will develop culturally appropriate information resources so that they are accessible, appealing, and empowering for Aboriginal people with arthritic conditions to allow better self-management of their symptoms.

### Arthritis and disparities in health care

Arthritis is an umbrella term used to identify a collection of over 100 musculoskeletal conditions, including osteoarthritis, rheumatoid arthritis, lupus and gout [10]. These conditions are leading causes of pain and disability, impacting 15% of the population or 3.6 million Australians [10]. Compared to the general population, people 45 years or older who experience arthritis are twice as likely to describe their health as poor, and twice as likely to report very high levels of psychological distress and also report experiencing moderate to very severe pain [10]. Like many chronic conditions, the impact of arthritis is heightened in populations that experience greater burden of disease as a result of health inequity, such as Indigenous populations [11].

### Impact of arthritis on Aboriginal communities

Arthritis is highly prevalent in Indigenous communities in Australia, Canada, New Zealand and the United States of America [5–7, 12]. In Australia, the prevalence of osteoarthritis in Aboriginal people is between 1.2 and 1.5 times higher than non-Aboriginal people [13] and the burden of disease is greater (31 Disability-Adjusted Life Years [DALY] per 1000 people for Aboriginal people versus 22 per 1000 people for non-Aboriginal people) [14]. The prevalence of lupus in Aboriginal people is 52.0–92.8 cases per 100 000 population compared to 19.3–39.0 cases per 100 000 population for non-Aboriginal people [15]. The burden of rheumatoid arthritis in Aboriginal people is 4.8 DALY per 1,000 people compared to 3.5 DALY per 1,000 for non-Aboriginal people [14].

Despite an increased prevalence and burden of arthritis within Aboriginal communities, the utilisation of care for arthritis is low. For example, Aboriginal people with hip or knee osteoarthritis access primary care services at half the rate based on incidence [16, 17]. Cultural barriers for Aboriginal people when accessing health care have been recognised since the 1970s [18] yet, Aboriginal people continue to report feeling unsafe, experience racism and difficulties in understanding or being understood by service providers [19, 20]. For Aboriginal people seeking care for arthritis, ineffective communication with health practitioners, lower levels of health literacy and experiences of stigma are further barriers to care [5]. One way to address these barriers is through improving access to culturally appropriate information tailored to the needs of Aboriginal people [5, 21, 22]. This is an essential step in building an accessible, culturally secure program of arthritis care that is required to address the current higher prevalence of arthritic conditions experienced by Aboriginal people.

### Evidence based management of arthritis and the role of patient information resources

Patient education and information resources such as booklets, factsheets and websites can be stand-alone or provided within health care consultations to reinforce verbal teaching and facilitate meaningful communication [23]. They aim to educate patients, families, and the public in an effort to increase health knowledge [24, 25] and in turn improve health literacy [26] and comprehension [27]. In doing so, patient education and information resources empower patients and their families to have greater involvement in decisions about care, to engage in self-management [28, 29] and adhere to medication and physical activity programs [30], positively impacting health outcomes [31, 32].

Peak national bodies such as Arthritis Australia, and State and Territory based arthritis organisations such as Arthritis and Osteoporosis Western Australia, provide an extensive range of arthritis management resources such as information sheets and booklets, patient education tools and programs and support services. Typically, these resources address beliefs and knowledge about arthritis and encourage patients to proactively self-manage their condition through pain management strategies, staying active and engaging in exercise and physical activity. Despite the availability of such resources, there are currently no arthritis resources that have been developed for Aboriginal people or involving input from Aboriginal consumers who experience arthritis. Aboriginal people need to be involved in the development of resources, as governance of the process is more likely to lead to resources which are meaningful and relevant to Aboriginal people with arthritis. This is likely to increase uptake of advice and recommendations made within the resources [9, 33].

## The Staying Moving, Staying Strong project

This paper outlines research that will be undertaken within the *Staying Moving*, *Staying Strong* project, an implementation project that aims to develop, implement and evaluate culturally secure arthritis information for Aboriginal people with osteoarthritis, rheumatoid arthritis, lupus and gout. This protocol relates to the first phase of research in which we will co-develop arthritis resources with community members through a six-stage study involving a systematic review and robust qualitative research methods. The outcome will be culturally secure information resources ready for implementation.

## Materials & methods

The overarching principle underpinning this project is cultural security, referring to the incorporation of processes such that the research will not compromise the cultural rights, values and expectations of Aboriginal people [3]. Such processes include privileging Aboriginal voices, and multilevel and ongoing Aboriginal community engagement to guide methodological and cultural aspects of this project. This will ensure responsivity to community needs as they are identified. We will also build the capabilities of Aboriginal researchers and clinicians to work in musculoskeletal health and non-Aboriginal researchers and clinicians to work within culturally secure research paradigms.

In this six-phase project (see Table 1), we will adopt a qualitative approach using the culturally secure research method of research *yarning* [34]. Senior researchers in the project team with expertise in research yarning will provide training to all researchers who will be conducting research yarns. Research yarning is acknowledged as a culturally secure approach to qualitative interviews as it privileges the lived experience and cultural context of the participants [35]. Research yarning aligns with Aboriginal ways of knowing and doing, such as the use of storytelling, and ensures interviews and focus groups are informal, relaxed and requires the researcher to build a relationship that is accountable to Aboriginal people participating in the research [34]. The yarning process is made up of difference forms of yarning. In the first phase and prior to the research yarn, the interviewer and participant engages in an unstructured, informal social yarn which aims to develop trust and rapport. Following the social yarn, the researcher may then engage in the research topic yarn, which aims to collect data through the participants’ stories within a semi-structured interview format [34]. Data collection and analysis will also be carried out concurrently by Aboriginal and non-Aboriginal researchers working alongside each other.

**Table 1:** Overview of project activities.

Phase	Objective	Project activities
Phase 1	To establish a community reference group and advisory group.	• Establish community reference group comprised of a diverse group of Aboriginal people who experience arthritis. • The community reference group will provide governance and oversight of the project activities, to advise on the strategic direction, cultural security, interpretation and dissemination of the research findings. • Establish an advisory group comprised of Aboriginal health researchers, health service providers, clinicians and health service researchers, Aboriginal consumers, and stakeholders. • The advisory committee will provide guidance on the methodological aspects of the study.
Phase 2	To explore the health information needs and preferences of Aboriginal people with arthritis.	• Qualitative study involving interviews with Aboriginal participants who have osteoarthritis, rheumatoid arthritis, lupus and gout. • Interviews explore participants’ experience of engaging with health resources, their informational needs in relation to arthritis and preferences regarding the format of health information. • Inclusion criteria: Aged 18 years or older, identify as Aboriginal and/or Torres Strait Islander, experience one or more of osteoarthritis, rheumatoid arthritis, lupus and gout, English speaking, able to provide informed consent and willing to participate. • Exclusion criteria: Requires an interpreter to participate, experience cognitive impairment preventing participants from providing meaningful responses to interview questions, psychological illness that may put researchers or interviewers at risk of harm.
Phase 3	To identify and synthesise key recommendations from high quality clinical practice guidelines on arthritis care.	• Literature review to identify contemporary clinical practice guidelines on osteoarthritis, rheumatoid arthritis, lupus and gout. • Quality appraisal of clinical practice guidelines using the Appraisal of Guidelines for Research Evaluation II (AGREE II) instrument. • Synthesis of key recommendations from high quality clinical practice guidelines.
Phase 4	To culturally adapt key recommendations.	• Key recommendations culturally adapted for Aboriginal people through consultation with the advisory group using a modified nominal group technique.
Phase 5	To develop culturally adapted arthritis resources for Aboriginal people.	• Draft arthritis resources will be developed based on the feedback received in phase 4. • Draft resources will be presented to the community reference group using a culturally adapted nominal group discussion. • Resources further adapted based on feedback.
Phase 6	To qualitatively evaluate the culturally adapted arthritis resources.	• Qualitative evaluation using research yarning and adapted think aloud exercises with Aboriginal consumers with osteoarthritis, rheumatoid arthritis, lupus and gout. • Inclusion criteria: Aged 18 years or older, identify as Aboriginal and/or Torres Strait Islander, experience one or more of osteoarthritis, rheumatoid arthritis, lupus and gout, English speaking, able to provide informed consent and willing to participate. • Exclusion criteria: Requires an interpreter to participate, experience cognitive impairment preventing participants from providing meaningful responses to interview questions, psychological illness that may put researchers or interviewers at risk of harm.

### Ethical considerations

This project follows the Australian National Health and Medical Research Council (NHMRC) guidelines for ethical conduct for research in Aboriginal and Torres Strait Islander People and communities and has been designed with multilevel Aboriginal engagement and consultation. Each of the six core values of spirit and integrity, cultural continuity, equity, reciprocity, respect, and responsibility have been addressed in ethics applications approved by the Western Australian Aboriginal Human Ethics Committee (HREC1018) on the 6^th^ of October 2020 and the St Vincent’s Hospital Melbourne Human Research Ethics Committee (HREC 224/20) on the 4^th^ of November 2020. These applications have also addressed the NHMRC Indigenous research excellence criteria: community engagement, benefit, sustainability and transferability and building capabilities.

### Patient and public involvement

This project is predicated on meaningful patient and public involvement. We will establish a community reference group and advisory group, both of which will comprise of a diverse group of Aboriginal consumers who experience arthritis. The community reference group and advisory group will have a significant role in guiding the conduct of all phases of this research.

### Partnerships

This project involves a national partnership between Arthritis and Osteoporosis Western Australia and the Enhancing Equity, Collaboration and Culturally secure Osteoarthritis care for Aboriginal Australians (ECCO) collaboration, with the support of Arthritis Australia and funding from the Commonwealth Government. The ECCO collaboration is an inter-professional team of Aboriginal and non-Aboriginal health practitioners, health service staff, and research leaders from the University of Melbourne, Geraldton Regional Aboriginal Medical Service, Victorian Aboriginal Heath Service, University of Western Australia, Western Australia Centre for Rural Health, Telethon Kids Institute, and St Vincent’s Hospital, Melbourne. ECCO also has established links with local Aboriginal Medical Services (AMSs) in Western Australia and Victoria. This collective was established with support from the NHMRC Centre for Research Excellence (CRE) in Total Joint Replacement which aims to OPtimise oUtcomes, equity, cost effectiveness and patient Selection for people with osteoarthritis (OPUS). Work conducted at the OPUS CRE identified that there is an absence of opportunity for Aboriginal people to access culturally appropriate arthritis information and culturally secure clinical care. The ECCO collaboration was formed to address this gap in musculoskeletal health research and to build an evidence-based model of arthritis care that addresses the needs of Aboriginal people.

### Participants

Participants and participant groups have been described within each of the six phases of this study outlined below.

## Methodology and analysis

### Phase 1: Establish a community reference group and advisory group

The first phase of this research will be to establish a community reference group and advisory group.

#### Community reference group

To recruit the community reference group, we will draw on our previously established partnerships and networks to identify Aboriginal community members who experience osteoarthritis, rheumatoid arthritis, lupus and/or gout. We will then use ‘snowballing’ recruitment to identify suitable community reference group members. We will leverage relationships rather than a formal admission interview process. A face to face or phone yarn will screen potential group members for suitability. Suitable candidates will be willing to participate and comfortable to share opinions and experiences in a group situation. Each arthritic condition, women and men of a range of ages and from different geographical locations (urban, regional, remote Australia) will be represented in the group. The main purpose of the community reference group is to provide governance and oversight of the project activities, to advise on the strategic direction, cultural security, interpretation and dissemination of the research findings. The group will meet bi-annually face-to-face and through a teleconferencing platform where required.

#### Advisory group

The advisory group will comprise of Aboriginal health researchers, health service providers, clinicians (including a rheumatologist), health service researchers, Aboriginal consumers, and stakeholders. Similarly, we will draw on our existing partnerships and research networks to recruit approximately 12 individuals. The primary aim of this group will be to advise on methodological aspects of the study and will also meet bi-annually through a teleconferencing platform.

### Phase 2. Explore the health information needs and preferences of Aboriginal people with arthritis

In the second phase of research we will conduct a qualitative study to explore the health information needs and preferences of Aboriginal people with arthritis. To recruit participants, we will draw on the network of investigators and existing partnerships with local AMSs. Potential participants will be provided a detailed Participant Information Sheet and an Aboriginal research assistant will follow up with phone call to further describe and discuss the study. The Aboriginal research assistant will answer any questions the potential participants may have and seek verbal consent once they are satisfied the participant is fully informed. Participants will be adults over the age of 18, as approaches to providing health information to children would likely differ significantly to the adult population. We will ensure that the sample comprises of a representative number of people with osteoarthritis, rheumatoid arthritis, lupus and gout, and that gender is diversely represented relative to the prevalence of each condition. We will employ maximal variation sampling to ensure that a range of ages and people from diverse geographical locations are included. Data collection and analysis will be conducted in parallel. Through our yarning approach, we anticipate that each interview will generate rich data. The sample size will be guided by thematic saturation within each group i.e. the point at which no new themes are emerging from the data. To capture a range of voices across diverse urban, regional and rural settings, we will aim to recruit 4–5 participants per condition at each of the two main study sites (Geraldton, Western Australia and Melbourne, Victoria). Thus, we anticipate recruiting approximately 8–10 participants per condition; for a total of 32–40 across all of the included conditions. Individual interviews held over a teleconferencing platform will be conducted by both an Aboriginal researcher and non-Aboriginal researcher with expertise interviewing people with arthritis. Researchers conducting the interviews will seek additional verbal informed consent, ensuring the participant has read and understood the Participant Information Sheet. Verbal consent from both participants and any family members present will be audiotaped prior to the beginning of the interview. Interview questions will explore participants’ experience of engaging with health resources in the past, their informational needs in relation to arthritis and preferences regarding the format of health information. Interviews will be audio-recorded and transcribed. Data will be analysed for each condition separately. Thematic analysis [36] of interview transcripts will be led by the two interviewers. To test/challenge emerging interpretations, preliminary findings from the analysis will be discussed with the community reference and advisory group. The outcome will be a set of recommendations about the content and format of health information based on the needs and preferences of Aboriginal people with arthritis.

### Phase 3. Synthesise key recommendations from high quality clinical practice guidelines on arthritis care

Adopting a methodology previously described in Lin et al. 2018 [37]; we will undertake a review of the literature and synthesise findings from contemporary clinical practice guidelines on osteoarthritis, rheumatoid arthritis, lupus and gout to identify recommendations related to patient information (See Open Science Framework Protocol Registration: DOI 10.17605/ https://osf.io/UB3Y7/). We will select clinical practice guidelines published within the last five years to reflect up-to-date research evidence. Quality of the retrieved clinical practice guidelines will be appraised using the Appraisal of Guidelines for Research Evaluation II (AGREE II) instrument [38]. The AGREE II has been widely used to assess the quality of clinical practice guidelines, with validated cut-off scores for high and low quality. Only clinical practice guidelines scored as high quality on the AGREE II will be included in our synthesis. Data will be synthesised separately for each condition. The synthesis will involve four stages: extracting information; classifying recommendations; developing a narrative summary and describing common recommendations relating to patient information.

### Phase 4. Cultural adaption of key recommendations

Key recommendations will be culturally adapted through consultation with the advisory group using a modified nominal group technique [39], a method for arriving at consensus among a group of experts. Members of the advisory group will be proved a detailed Participant Information Sheet and be given an opportunity to ask questions about participating before being involved in the nominal group discussion. All participants will sign a written consent form on the day of the nominal group discussion. The modified nominal group technique will begin with a yarning session about the key recommendations identified in phases 2 and 3. The advisory group will be asked a question such as: "How can we adapt these key recommendations for Aboriginal consumers?" Participants will be encouraged to consider both the content and format of the recommendations. Following this, in a round-robin fashion, each participant will be invited to present one response or idea at a time. The ideas will be recorded on a whiteboard or virtual whiteboard made visible to all participants. This phase will continue until no new ideas are generated. Participants will then discuss and clarify the ideas before grouping ideas together into themes. In the final step, themes will be ranked and prioritized. Participants will be invited to vote for the themes they deem most important. A rank-order will then be created for listed themes based on total votes. The outcome will be a set of culturally informed and adapted arthritis recommendations.

### Phase 5. Develop culturally adapted arthritis resources

Based on the outcomes of phase four, the researchers will draft a cultural adaption of the key recommendations and format them in a way that meets the needs of Aboriginal consumers and is informed by evidence-based frameworks for the development of patient health information [40, 41]. This may take the form of print, video or web-based materials or a combination of all. Principles such as observing, watching and narrative storytelling may be incorporated into the formats chosen and will be guided by the preferences of the participants. A first draft of the resources will be presented to the community reference group using a culturally adapted nominal group discussion as outlined in phase 4. Members of the community reference group will be proved a detailed Participant Information Sheet and be given an opportunity to ask questions about participating before being involved in the nominal group discussion. All participants will sign a written consent form on the day of the nominal group discussion. First, the community reference group members will be given the opportunity to interact with the draft resources. Following this, a structured yarning discussion will be facilitated to determine how well the resources meet the needs of Aboriginal consumers with arthritis. Key themes will be summarised and ranked and the resulting recommendations will be used to refine the resources.

### Phase 6. Qualitative evaluation of resources

We will conduct a qualitative evaluation of the resources among Aboriginal consumers. The sample will include a representative number of people with osteoarthritis, rheumatoid arthritis, lupus and gout and men and women who represent a range of ages and come from diverse geographical locations across urban, regional, remote Australia. While the actual sample size will be guided by thematic saturation, we will aim to recruit approximately 6–8 individuals per condition. Recruitment will be facilitated through existing networks and AMSs at each study site and the same informed consent procedures adopted in Phase 2 will be used to ensure participants are fully informed before participating in the evaluation. Employing culturally appropriate research yarning, the qualitative evaluation will involve an adapted think aloud exercise [42] and semi-structured interviews. In the think aloud exercise, participants will be asked to review the culturally adapted resources and ’think aloud’ about what they are experiencing while being observed by the interviewer. The interviewer will prompt the participant during the observation such as "Can you describe what you are seeing to me?" and "How does what you are seeing make you feel?" to encourage participants to share their understanding and reactions to the resources. Researchers will pay particular attention to participants non-verbal cues, prompting participants to clarify their behaviour and thought process, for example: “What were you thinking when you stopped at…?” The think aloud exercise will be followed by a research yarn to explore how key messages in the resources were taken up by the participants in light of the participants’ underlying assumptions (i.e. how the participants pre-existing experiences interact with what parts of the information they give attention to, accept or dismiss). We will consider not only the intended effects of the messages (i.e. on the attitudes, beliefs, intentions of the participants) but also any potential unintended effects of the messages (e.g. on the physical, mental, social and cultural wellbeing of the participants). Data will be collected and analysed in parallel so that emerging interpretations can be tested and challenged in subsequent interviews. Data will be analysed using a critical approach [43] and the outcome will be categorised into themes describing the participants’ experiences of engaging with the culturally adapted arthritis resources.

## Data management

All data collected in this project including audio recordings of interviews and focus groups will be stored on a password protected computer kept on secure servers and in a locked facility at University of Melbourne and the University of Western Australia. Only project investigators as approved by the Western Australian Aboriginal Health Ethics Committee and St Vincent’s Hospital Melbourne Human Research Ethics Committee will have access to primary data. Transcription of audio data will be outsourced to a trusted Australian transcription company who adhere to strict confidentiality agreements and the Commonwealth Privacy Act. These transcripts will not record any identifying information.

## Timelines

Data collection for this project will commence in the first quarter of 2021 with an overall timeline described in Table 2.

**Table 2:** Project timelines.

	Year 1				Year 2			
Milestone	Q1	Q2	Q3	Q4	Q1	Q2	Q3	Q4
**Project planning:** Set up team communication plan & progress meetings								
**Project planning:** Recruitment of additional Aboriginal and Torres Strait Islander staff in WA & VIC								
**Project planning**: Develop project implementation document								
**Phase 1:** Terms of Reference for advisory committees								
**Phase 1:** Convene Executive Advisory Group and Community Reference Group								
**Phase 2:** Qualitative investigation of health information needs								
**Phase 3:** Systematic review of existing arthritis resources								
**Phase 4:** Modification of key recommendations with Advisory Group								
**Phase 5:** Draft arthritis resources								
**Phase 5:** Further cultural adaptation of draft resources with Community Reference Group								
**Phase 5:** Final resources developed								
**Phase 6:** Qualitative evaluation of final resources								

## Project outcomes

The output will be culturally appropriate health information resources for Aboriginal people with arthritis that can be used in health care and self-management, or as stand-alone consumer information resources.

## Discussion

In this project, culturally secure processes will be employed in each step of the development of patient education and information materials to ensure that they are culturally appropriate and relevant to Aboriginal consumers with arthritis. The focus of this research is osteoarthritis, rheumatoid arthritis, lupus and gout, in line with the funding scheme. While other musculoskeletal conditions are beyond the scope of this project, the outcomes of this research will have relevance to musculoskeletal conditions more broadly. Adopting culturally secure research methods which prioritise partnerships, community engagement, community benefit, sustainability and transferability and capacity building, the framework we have presented in this protocol paper may also be applied by other groups seeking to develop culturally secure resources in other areas of health.

Foundational to this project will be the formation of a diverse and representative expert advisory committee and Aboriginal community reference group. The advisory group and community reference group will oversee project activities, steering both methodological and cultural aspects of the project, therefore allowing the research team to respond to community identified priorities. Addressing the burden of musculoskeletal health conditions in Aboriginal communities is currently considered an unmet health need [5]. This project will generate new knowledge regarding the lived experience of Aboriginal people with arthritis as well as how Aboriginal people engage with arthritis information resources. This is an essential step to improve patient-practitioner communication within health services and may improve the health outcomes for Aboriginal people living with arthritis [14].

To ensure the sustainability of this project, this team will build on existing partnerships with AMSs, Aboriginal Community Controlled Health Services and major acute health services. Such partnerships will be important sources of dissemination, distribution, and translation of the developed resources into the clinical environment. Key learnings may also be transferable to the development of resources for conditions other than arthritis. By working closely with health services, we will build the capabilities of Aboriginal and non-Aboriginal clinicians to respond to and manage joint pain and arthritis. Further to this, capacity building within the research team is a key element of this project. We will employ two part-time Aboriginal research assistants and appoint an Aboriginal PhD student who will be mentored by experts in musculoskeletal health research and Aboriginal health research.

There are potential limitations in relation to the scope of this study. Firstly, we will only include English speaking participants. More than 150 Aboriginal languages were recorded as spoken at home in Australia in 2016 and many Aboriginal people speak English as an additional language [44, 45]. Although initially only English-speaking participants will be included, the resources may be translated into some Aboriginal languages if recommended by the Aboriginal consumers included in this project. Audio-visual formats will also be available in the development of the resources, as observing/watching may be a more culturally appropriate form of learning than reading. Secondly, the authors acknowledge limitations associated with employing a qualitative evaluation methodology. While inferences made through the testing and evaluation of the developed resources will be limited in their scope, this project provides a robust foundation for which future research can be based upon. Future directions for related research could include a randomized controlled trial to test the efficacy of culturally adapted resources on the knowledge, beliefs, health behaviours and health-related outcomes of Aboriginal people who experience arthritis.

## Conclusions

This project outlines one aspect of addressing disparities in musculoskeletal health care available for Aboriginal people. Patient education and information resources can be a powerful tool to address communication challenges, health literacy and health care access for Aboriginal people who experience arthritis and to empower people to be able to self-manage their conditions. Embedded in a larger body of work seeking to develop a culturally secure model of arthritis care, the Staying Moving Staying Strong project will benefit over 85,000 Aboriginal people who are currently living with arthritis in Australia.

## Abbreviations

AGREE II: Appraisal of Guidelines for Research Evaluation II instrument
AMS: Aboriginal Medical Service
CRE: Australian National Health and Medical Research Council Centre for Research Excellence
DALY: Disability-Adjusted Life Years
ECCO: The Enhancing Equity, Collaboration and Culturally secure Osteoarthritis care for Aboriginal Australians collaboration
Lupus: Systemic lupus erythematosus
OPUS: Centre for Research Excellence in Total Joint Replacement—OPtimise oUtcomes, equity, cost effectiveness and patient Selection for people with osteoarthritis
NHMRC: Australian National Health and Medical Research Council
